# Metabolomic alternations of follicular fluid of obese women undergoing *in-vitro* fertilization treatment

**DOI:** 10.1038/s41598-020-62975-z

**Published:** 2020-04-06

**Authors:** Jingyan Song, Shan Xiang, Conghui Pang, Jiayin Guo, Zhengao Sun

**Affiliations:** 1 The First Clinical College, Shandong University of Traditional Chinese Medicine, Jinan, 250014 China; 2 Reproductive and Genetic Center of Integrated Traditional and Western Medicine, The Affiliated Hospital of Shandong University of Traditional Chinese Medicine, Jinan, 250011 China; 3 Guangdong Provincial Key Laboratory of New Drug Screening, School of Pharmaceutical Sciences, Southern Medical University, Guangzhou, 510515 China

**Keywords:** Systems biology, Systems biology, Medical research, Medical research

## Abstract

Obesity exerts negative effects on the metabolic homeostasis of cells in various tissues, but how it influences ovum metabolism is not fully understood. Previous studies demonstrate that oocyte genes that regulate oxidative stress, lipid metabolism, and inflammation are highly expressed in obese women. However, the metabolic effects of these genetic variations are not clear. To address this gap, we conducted an exploratory evaluation of follicular fluid (FF) metabolites in underweight, normal-weight, overweight, and obese women undergoing *in vitro* fertilization (IVF) treatment. The FF samples from the underweight (Group A, n = 40), normal-weight (Group B, n = 40), overweight (Group C, n = 40), and obese women (Group D, n = 40) were analyzed using ultra-performance liquid chromatography high-resolution mass spectrometry. A novel, high-coverage, semi-targeted metabolomics method (SWATH to MRM) and a targeted metabolomics method were employed to identify and verify the differential metabolites between the four groups. Sixteen differentially expressed FF metabolites were identified. Increase of BMI was associated with upregulation of 5 metabolites, ganoderiol H, LPI (18:3), sedoheptulose 1,7-bisphosphate, austalide L and 2 - {[hydroxyl (3-hydroxy-4-methoxyphenylmethylidene] amino} acetic acid, and downregulation of 5 metabolites, 1-phenyl-1,3-elcosanedione, retinol acetate, p-Cresol sulfate, setariol and arachidonyl carnitine. These metabolites were enriched in different metabolic pathways of retinol metabolism and fatty acid metabolism. These obesity-related differential metabolites provide a pathogenesis mechanism that explains the decline of oocyte development during obesity. These results suggest that obesity affects follicular environment prior to pregnancy, a time-window that may be important for lifestyle interventions to decrease obesity levels.

## Introduction

Antral follicles in mammalian ovaries are composed of oocytes, one or more layers of granulosa cells and outer membrane cells. Oocytes bind to granulosa cells through gap junctions, which mediate the transfer of small metabolites, inorganic ions, and second messengers from one cell to another to supply nutrients and growth factors that promote oocyte growth, development, and maturation^1,2^. Oocyte development is a complex process regulated by many internal and external factors in the ovary. Granulosa cells provide a variety of energy substrates for oocytes while oocytes control the metabolic activities of granulosa cells by secreting paracrine factors^2,3^. During oocyte maturation, a number of metabolites and metabolism-related enzymes have been demonstrated to play important roles in various cellular events^1,4–6^. In the last four decades, the wide-spread application of IVF-ET technology has not only brought joy to the majority of infertility patients, but also provided a platform for studying the development of oocytes and improve their ontogenetical potential. Moreover, about half of women of child-bearing age undergoing assisted reproductive technology (ART) are overweight or obese. Therefore, it is important to understand the impact of overweight on oocytes development^7,8^.

Meanwhile, obesity and its related metabolic disorders cause major health problems that have attracted global attention. In particular, several studies have confirmed that obese women have a significantly increased risk of infertility, abortion, obstetric complications, neonatal morbidity, and mortality, and birth defects^9–15^. Previous studies have revealed that obese women undergoing IVF treatment have decreased number and quality of oocytes and embryos in comparison to normal-weight women^16–18^. Another recent study found that the expression of genes related to inflammation, oxidative stress, lipid metabolism and transcription factors in oocytes of overweight or obese women was statistically significant compared to those of women with normal weight^19^. These results suggest that obesity changes in the microenvironment of the oocytes before pregnancy and this is likely to affect the pregnancy outcome. These results were obtained by studying the medium follicular fluid (FF), and thus analysis of FF composition provides a unique opportunity to assess factors such as the oocyte environment at ovulation and the metabolic information of oocytes and surrounding granulosa cells. Recent clinical studies have shown that in obese women undergoing fertility treatment, FF triglyceride, leptin, and c-reactive protein levels are elevated and these are positively correlated with body mass index (BMI), poor oocyte quality, and pregnancy outcome^19,20^. Collectively, these studies demonstrate the importance of FF in providing a favorable follicular environment for a successful pregnancy, especially in obese patients. Recently, untargeted metabolomics has been widely used to detect metabolites in FF, enabling us to understand how the surrounding metabolites affecting oocyte development^21–24^. However, no comprehensive untargeted metabolomics analysis has been done to examine the impact of obesity on human FF metabolites. This experimental design study investigated the underlying mechanisms of follicular metabolomics in obese women undergoing IVF.

## Materials and Methods

### Participants

The MetSizeR approach was used to estimate the sample size of 40 participants based on the following assumptions^23–28^: mass spectrometry of 584 follicular fluid metabolites, a target false detection rate of 5%, and an expected proportion of significant metabolites of 20%^29^. A total of 160 subjects were recruited and their FFs collected at the affiliated hospital of Shandong University of Traditional Chinese Medicine, from November 2017 to May 2018. The subjects were divided into four groups; group A (BMI < 18.5 kg/m^2^, n = 40), the group B (18.5 kg/m^2^ ≤ BMI < 25 kg/m^2^, n = 40), the group C (25 kg/m^2^ ≤ BMI < 30 kg/m^2^, n = 40) and group D (BMI ≥ 30 kg/m^2^, n = 40). The recruited subjects were included or excluded in our study according to the inclusion and exclusion criteria.

### Ethical considerations

All experiments were performed in accordance with institutional guidelines and received approvals from the Health Authorities and Ethics Committees of the Affiliated Hospital of Shandong University of Traditional Chinese Medicine. All participants carefully read and appended their signatures on the informed consent forms prior to the commencement of the study.

### Inclusion and exclusion criteria

Inclusion criteria: (1) all patients receiving IVF treatment due to fallopian tube associated problems; (2) healthy women aged between 21–35 years.

Exclusion criteria: (1) women who had endometriosis, polycystic ovary syndrome (PCOS), genital abnormalities, chronic hypertension, diabetes, autoimmune diseases, infectious diseases, or liver, kidney, cardiovascular, or thyroid diseases; (2) subjects aged ≥ 40 years old.

### FF retrieval procedures

GnRH antagonist (cetrorelix; Merck Serono, Darmstadt, Germany) is administered subcutaneously at a daily dose of 0.25 mg when there is at least one follicle measuring ≥12 mm in mean diameter on the trigger day, with 150–450 IU/day of recombinant FSH (Puregon, MSD, Courbevoie, France; Gonal-F, Merck-Serono, Lyon, France) and urinary FSH (hMG, Menotrophin for Injection, Livzon Pharmaceutical Group Inc, Guangdong, China). Gonadotropin doses will be determined based on individual patient’s characteristics. Final oocyte maturation will be triggered when more than two ovarian dominant follicles measuring ≥18 mm are visible by ultrasound. Final oocyte maturation will be achieved using either a single 0.2 mg injection of GnRH agonist (Triptoreline, Decapeptyl, Ipsen, France) or 250 μg of recombinant hCG (rhCG, Ovitrelle, Serono, France). Oocyte retrieval will be performed after 35–36 h by transvaginal ultrasound-guided aspiration. For each participant, the FF was collected from multiple mature follicles and pooled for each participant. After oocyte isolation, FF was centrifuged at 14,000 × g for 20 min to remove cells and insoluble particles. The supernatant was then transferred to sterile cryovials and stored at −80 °C for further analysis. Subsequently, an elective freeze-all strategy was performed and all the embryos were vitrified at cleavage stage on day 3.

### Sample preparation

200 μL of FF samples were mixed with 600 μL of methanol/isopropanol/water (4:4:2) containing six internal standards: d3-hexanoyl-carnitine, L-tryptophan-d5, d3-decanoyl-carnitine, PE (15:0/15:0), TG (15:0/15:0/15:0) and PC (17:0/17:0). The mixture was vortexed for 5 min and centrifuged at 14000 × g for 30 min, at 4 °C. The supernatant was transferred to an autosampler plate for analysis.

### LC-MS condition

A SCIEX ExionLC AD ultra-performance liquid chromatography (UPLC) system and a reverse-phase ACQUITY UPLC® BEH C18 column (2.1 × 100 mm, 1.7 μm) were used for metabolomics analysis. 5 μL of FF was injected at 15 °C and the total flow rate set at 0.4 mL/min. The column temperature was set at 40 °C. In positive mode, water with 0.1% formic acid (FA) served as mobile phase A and acetonitrile with 0.1% FA as mobile phase B. The elution gradient was maintained at 95% A for 0.5 min, it was then increased to 100% B over the next 7 min, and then returned to 95% A from 10 min to 10.1 min. The total running time was 12 min. In the negative mode, water containing 5 mM of ammonium acetate served as mobile phase A, and acetonitrile was used as mobile phase B. The elution gradient was kept at 95% A for 0.5 min, increased to 100% B over the next 8 min, and then returned to 95% A from 12 min to 12.1 min and the total running time was 14 min. All SWATH data were acquired using a SCIEX Triple TOF 5600+, and all MRM data were obtained on a SCIEX QTRAP 5500. The nebulizer gas (GS1 and GS2) was set at 55 psi and the source temperature was set at 550 °C.

**Figure 1:**
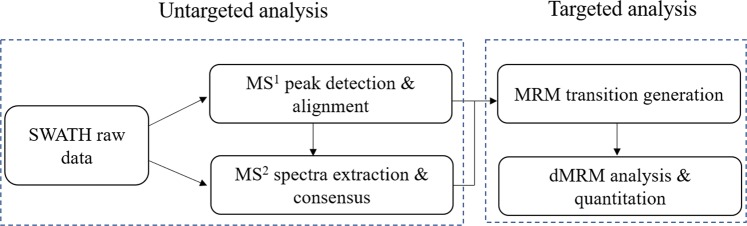
The workflow of SWATHtoMRM method.

In the positive mode, the voltage of ion spray was 5,500 V. The declustering potential and collision energy were set at 60 V and 35 ± 15 V, respectively. In the negative mode, the voltage of ion spray was −4,500 V. The declustering potential and collision energy were set at −60 V and −35 ± 15 V, respectively. The full scan range and the production scan range spun from m/z 50 to m/z 1200. The raw SWATH data were converted to mzXML files using the “msconvert” program from ProteoWizard. Multiple data files were grouped and processed with SWATHtoMRM to generate 584 MRM transitions^30^. The Analyst TF 1.7.1 software was used to maximize the number of measured MRM transitions in each analysis using the scheduled MRM method. The flow diagram of the SWATHtoMRM analytical process is provided in Fig. 1.

### Data processing and statistical analysis

Group differences among clinical variables were compared with one-way analysis of variance (ANOVA) and Chi-square (χ2) test. Scheffe was performed as ANOVA post hoc tests using SPSS software 22.0 (IBM Corp., USA). UPLC-TOF and UPLC-QTRAP were used to analyze 160 FF samples and this was done in three replicates. PeakView software was used for data processing while MarkerView software was used for peak detection, extraction of MS2 peaks and chromatograms, and MS1 and MS2 peak grouping. According to the “80% rule”^31^, peaks present in more than 80% of the samples of each group were chosen for further analysis. In large-scale metabolomic measurements, the reproducibility of the analyses were influenced by the source contamination or the maintenance and cleaning of the mass-spectrometer. Normalization is a common preprocessing method used to reduce systematic change. In this study, peak areas of all metabolites were normalized using internal standards. Only the metabolites with an RSD value below 15% in QC samples were used for statistical analysis. Principal component analysis (PCA) was employed to identify differential variation features on the MarkerView software. Univariate statistical analysis was performed with the Student’s t-test. Variables with univariate statistical significance (p < 0.05) were considered different among the four groups. In addition, supervised partial least-squares discriminant analysis (PLS-DA) was applied to model all features of the four groups in MetaboAnalyst. The predictability of the model was determined by internal validation using a 7-fold cross-validation and response permutation testing. The best-fitted PLS-DA models were used to explore the variable importance in the projection (VIP) value of all variables. Significance analysis of microarray (SAM) was performed to address the false discovery rate (FDR) for multiple tests. Differential variables with a VIP value greater than 1 and an FDR value less than 0.05 were selected. Potential differential variables were validated by p value, VIP value and FDR value. These differential variables were identified by accurate mass, isotope patterns, and mass spectrometric fragmentation patterns. They were further characterized using databases such as KEGG, PubChem compound, METLIN, the Madison Metabolomics Consortium Database, and the Human Database.

### Targeted metabolomics study

The UHPLC system (LC-30AD, Shimadzu) coupled to a Turbo V electrospray ionization source and a Qtrap 5500 mass spectrometer was used for targeted metabolomics analysis. MRM transitions were employed to perform MS detection. Sixteen metabolites were targeted in a single injection using both positive and negative modes with rapid polarity switching (50 ms). MultiQuant 3.0 (SCIEX) was used for data processing and MetaboAnalyst used for statistical analysis.

## Results

### Analytical characteristics of SWATHtoMRM method

To assess the reliability of the SWATH to MRM method, six compounds; d3-hexanoyl-carnitine, d5-L-tryptophan, d3-decanoyl-carnitine, PE (15:0/15:0), TG (15:0/15:0/15:0) and PC (17:0/17:0) were used as internal standards. A series of concentrations of internal standards were prepared and added to the FF sample. Six FF samples were prepared and analyzed in triplicate. The linear curve of each internal standard was constructed using its mean peak area at each concentration. The linear regression coefficients of d3-hexanoyl-carnitine, d5-L-tryptophan, d3-decanoyl-carnitine, PE (15:0/15:0), TG (15:0/15:0/15:0) and PC (17:0/17:0) were 0.9921, 0.9937, 0.9918, 0.9965, 0.9944 and 0.9953, respectively. These results revealed strong linear relationships.

Recovery was assessed at low, medium, and high concentrations for each internal standard. The mixture at each concentration was added to the FF matrix prior to the extraction or instrumental analysis. A recovery experiment was conducted in triplicate for quality control (QC) purposes. Recoveries were calculated using the peak area ratios of the standards spiked before extraction to the standard spiked before instrumental analysis. The results indicated that the recoveries ranged from 86.4% to 113.7% for the six internal standards at low, medium, and high concentrations. Therefore, the recovery rate of the detection method used was satisfactory.

The relative standard deviation (RSD) of the ratios of the peak numbers and peak areas of six QC samples were used to evaluate the repeatability. For metabolomics analysis, 89.2% peaks occurred at RSD < 15% and accounted for 95.4% of the summed responses in the positive mode, while 92.7% peaks occurred at RSD < 15% and accounted for 93.9% of the total responses in the negative mode.

### Participant characteristics

There were no differences recorded in any of the basic characteristics of the subjects in the four groups, including ovarian stimulation parameters (see Table 1).

**Table 1:** Basic characteristics, IVF laboratory parameters and pregnancy outcomes of recruited subjects in the present study.

Parameter	Group A (n = 40)	Group B (n = 40)	Group C (n = 40)	Group D (n = 40)	*p-value*
Age (years)^**a**^	28.40 ± 2.69	29.10 ± 2.73	29.35 ± 3.75	29.20 ± 3.21	*0.538*
BMI (kg/m^2^)^**a**^	17.63 ± 0.74	21.85 ± 1.97	26.91 ± 1.23	33.75 ± 2.30	***<0.001***
Infertility types (n, %)^**b**^					*0.763*
Primary infertility	16 (40%)	14 (35%)	14 (35%)	18 (45%)	
Secondary infertility	24 (60%)	26 (65%)	26 (65%)	22 (55%)	
Infertility duration (years)^**a**^	2.90 ± 1.87	3.15 ± 2.34	2.85 ± 1.33	3.75 ± 1.61	*0.109*
Basal FSH (U/L)^**a**^	7.55 ± 1.50	7.12 ± 2.12	7.49 ± 3.58	6.52 ± 1.89	*0.206*
Basal LH (U/L)^**a**^	6.09 ± 2.91	5.52 ± 1.48	4.85 ± 2.36	5.30 ± 2.50	*0.137*
Basal AFC (n)^**a**^	15.25 ± 4.13	17.05 ± 6.00	17.60 ± 5.36	16.95 ± 4.90	*0.201*
Gonadotropin dosage (U)^**a**^	2336.38 ± 797.95	2105.00 ± 571.39	2340.31 ± 977.94	2169.47 ± 639.38	*0.407*
Interval of COS (d)^**a**^	11.35 ± 1.33	11.65 ± 1.29	12.00 ± 1.22	12.23 ± 2.40	*0.088*
Retrieved oocytes (n)^**a**^	14.40 ± 6.11	15.85 ± 8.62	12.70 ± 5.65	14.45 ± 6.53	*0.236*
Metaphase α oocytes (n)^**a**^	11.05 ± 5.03	11.10 ± 4.86	9.35 ± 4.38	11.20 ± 5.94	*0.305*
2PN fertilized oocytes (n)^**a**^	9.30 ± 4.43	10.00 ± 4.30	7.90 ± 4.01	8.30 ± 4.10	*0.109*
Usable embryos (n)^**a**^	4.50 ± 2.94	5.70 ± 1.90	3.95 ± 2.80	4.15 ± 2.49	***0.012***
FET cycles (n)^**a**^	1.65 ± 1.17	1.80 ± 0.69	1.35 ± 0.74	1.35 ± 0.66	***0.038***
Clinical pregnancy rate (n, %)^**b**^	25/66 (37.9%)	30/72 (41.7%)	21/58(36.2%)	20/60 (33.3%)	*0.796*
Pregnancy loss rate (n, %)^**b**^	4/25 (16.0%)	4/30 (13.3%)	4/21 (19.1%)	5/20 (25.0%)	*0.753*
Live birth rate (n, %)^**b**^	21/66 (31.8%)	26/72 (36.1%)	17/58 (29.3%)	15/60 (25.0%)	*0.575*

COS, controlled ovarian stimulation; 2PN, two pronucleus; FET, frozen embryo transfer.

^**a**^One-way ANOVA analysis. ^**b**^Chi-square (${\rm{\chi }}2$) test.

In the IVF laboratory parameters, the BMI was similar while the number of usable embryos and FET cycles were different between groups. The number of oocytes and mature oocytes obtained from the four groups was similar. No differences were detected in the 2PN fertilized oocytes parameters (see Table 1). Notably, the number of usable embryos from overweight and obese women was smaller than those from women with normal BMI (p = 0.014 and p = 0.038, respectively). Similar results were obtained in the number of FET cycles (p = 0.03 and p = 0.02, respectively).

**Figure 2:**
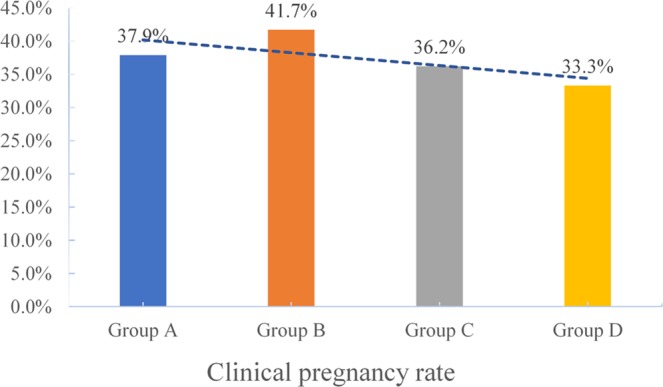
The clinical pregnancy rates of group A (underweight), group B (normal weight), group C (overweight) and group D (obesity)women undergoing IVF treatment.

**Figure 3:**
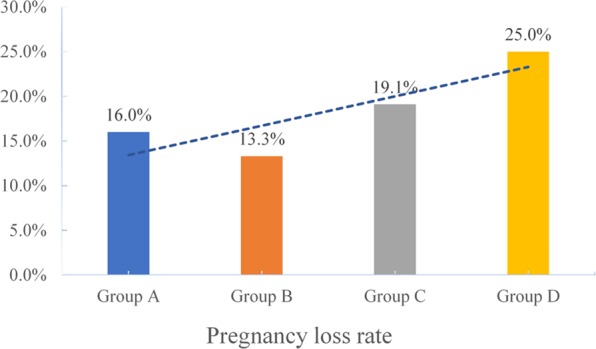
The pregnancy loss rates of group A (underweight), group B (normal weight), group C (overweight) and group D (obesity) women undergoing IVF treatment.

**Figure 4:**
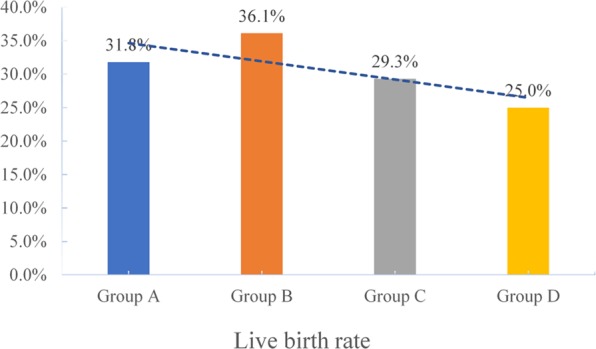
The live birth rates of group A (underweight), group B (normal weight), group C (overweight) and group D (obesity) women undergoing IVF treatment.

The clinical pregnancy rates (CPRs) were 37.9%, 41.7%, 36.2%, and 33.3% in the underweight, normal weight, overweight, and obese groups, respectively. The pregnancy loss rates (PLRs, biochemical pregnancies plus clinical pregnancies) were 16%, 13.3%, 19.1%, and 25% respectively in the four groups. The live birth rates (LBRs) were 31.8%, 36.1%, 29.3%, and 25% in the four groups, respectively. The study was underpowered to detect a difference in CPRs, PLRs, and LBRs, but there was a trend toward disadvantage as the BMI increased(see Figs. 2–4).

### Metabolites identification

Thousands of compound features in FF were obtained. The QTOF data were converted to mzXML files using the “msconvert” program from ProteoWizard. Multiple data files were grouped and processed by SWATH to MRM. A large-scale set of MRM transitions was produced, and a scheduled MRM method was performed. The top 16 metabolites (VIP > 1 and p < 0.01) were considered as the potential differential metabolites. The differential metabolites were identified based on their accurate mass, isotope ratio, and MS/MS spectra.

**Figure 5:**
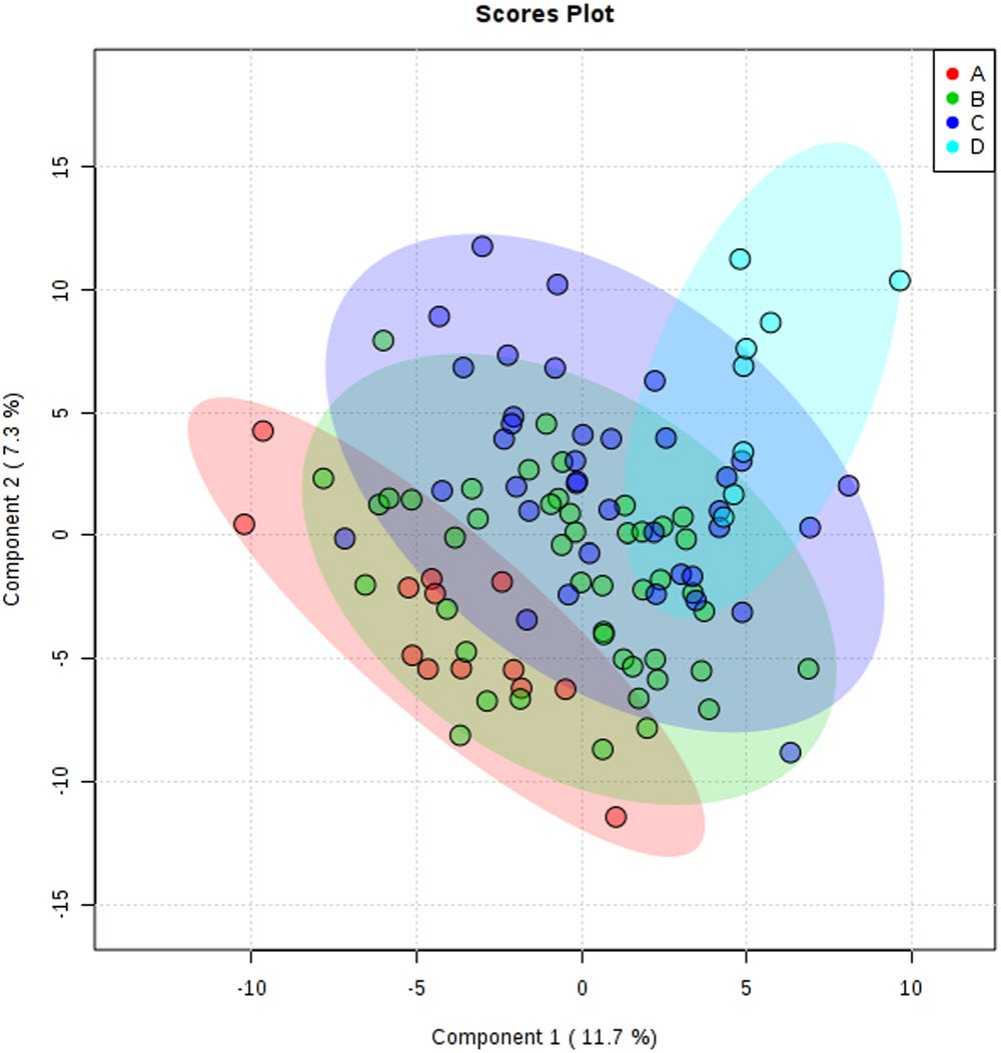
Two-dimensional score of follicular fluid samples of group A (underweight), group B (normal weight), group C (overweight) and group D (obese) women undergoing IVF treatment by PLS-DA analysis.

**Figure 6:**
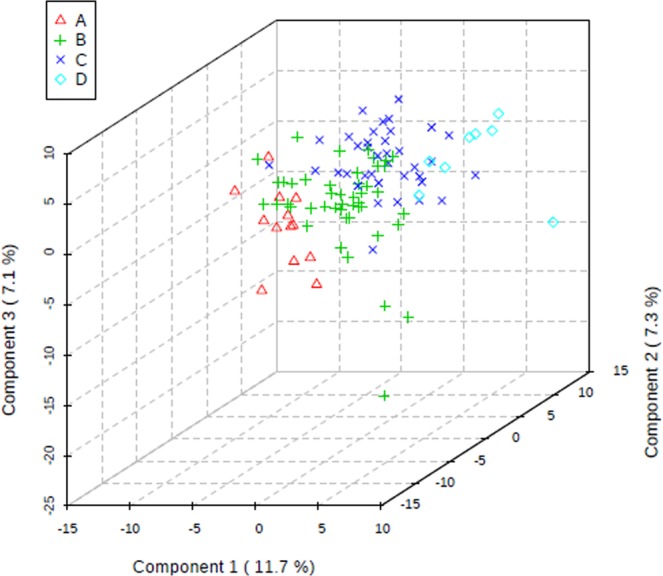
Three-dimensional score of follicular fluid samples of group A (underweight), group B (normal weight), group C (overweight) and group D (obese) women undergoing IVF treatment by PLS-DA analysis.

**Figure 7:**
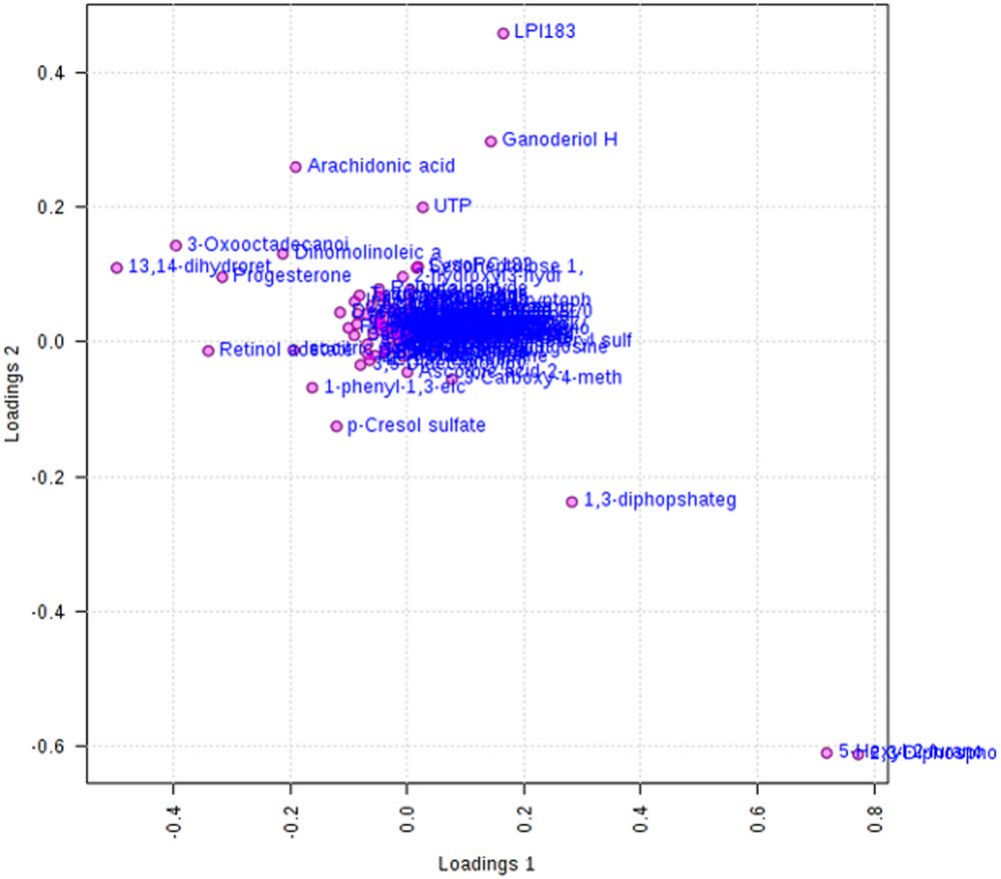
PLS-DA Loading plot in metabolomics analysis.

The study subjects were divided into four groups based on their BMI index (BMI < 18.5; 18.5 ≤ BMI < 25; 25 ≤ BMI < 30; BMI ≥ 30). The two-dimensional scoring chart (Fig. 5) shows that in the metabolomics analysis of FF samples, the samples gradually separated with the increase in BMI index. The three-dimensional scoring chart (Fig. 6) shows the distribution of each sample in three-dimensional space. Figures 5, 6 reveals that the samples in the four groups were well separated, and better separation was achieved at higher differences in BMI index. And, the scattering plots are obtained for the PLS-DA models on which Figs. 5, 6 was based (see Fig. 7).

**Figure 8:**
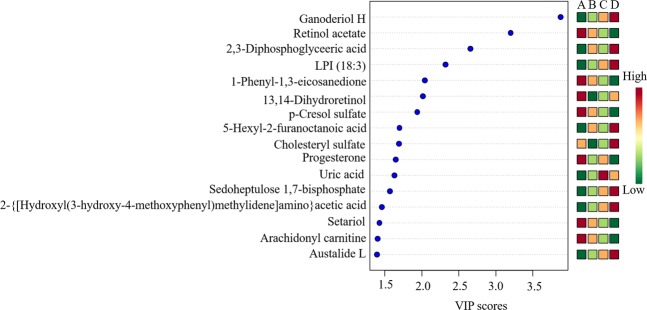
Differences in important follicular fluid characteristics between the four groups; group A (underweight), group B (normal weight), group C (overweight) and group D (obese) women undergoing IVF treatment (VIP scores).

**Figure 9:**
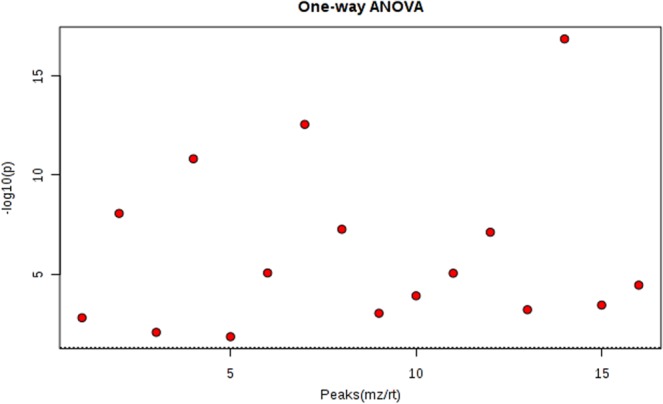
One-way analysis of variance (ANOVA) univariate statistics.

**Figure 10:**
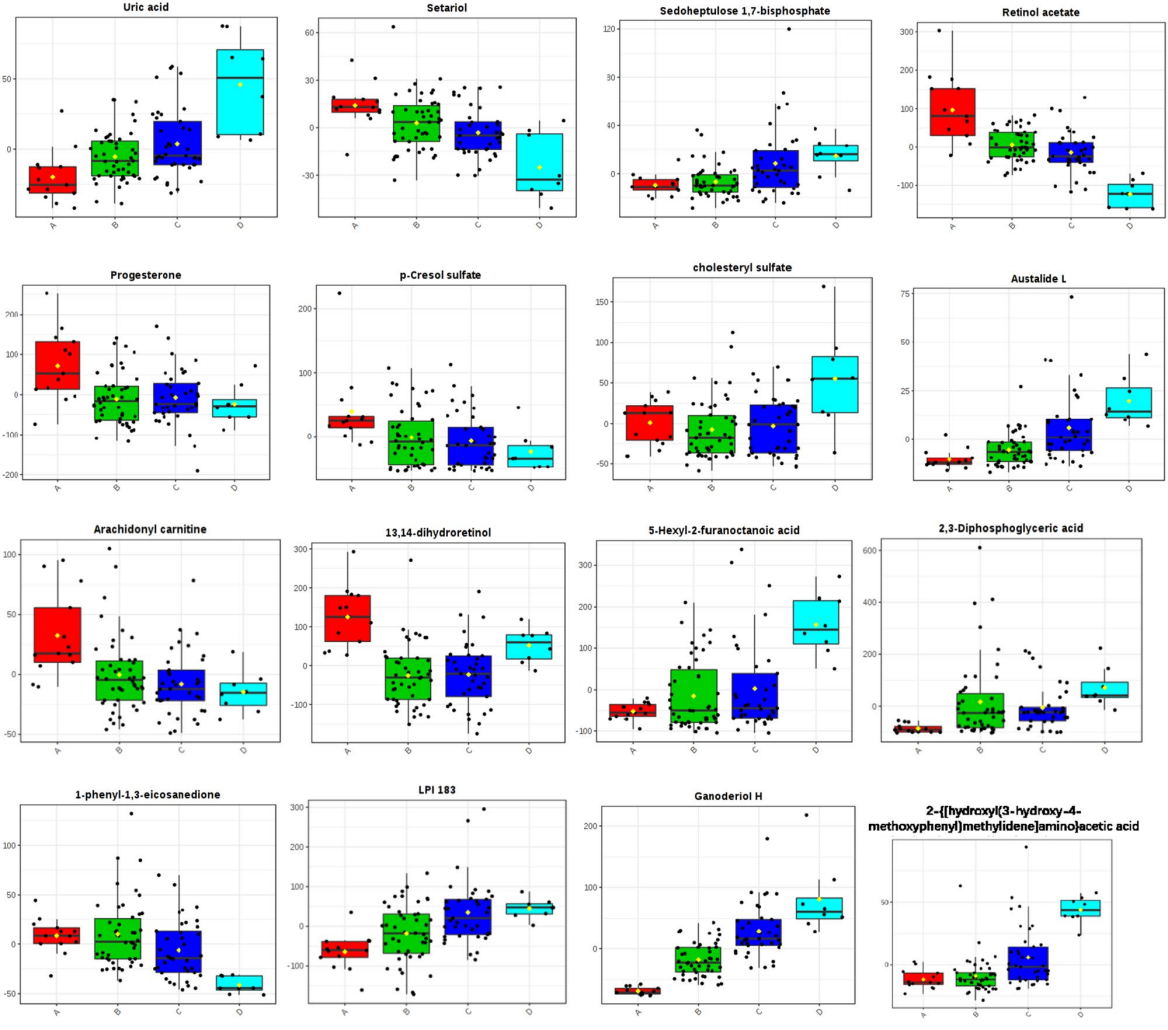
Metabolite profiles of the 16 candidate biomarkers (M1-M16) obtained from the quantitative analysis of the subjects.

In total, 16 differential metabolites were identified; ganoderiol H, retinol acetate, 2,3-diphosphoglyceric acid, LPI (18:3), 1-phenyl-1,3-elcosanedione, 13,14-dihydroretinol, p-cresol sulfate, 5-hexyl-2-furanoctanoic acid, cholesteryl sulfate, progesterone, uric acid, sedoheptulose 1,7-bisphosphate, 2- {[hydroxyl (3-hydroxy-4-methoxyphenyl) methylidene] amino} acetic acid, setariol, arachidonyl carnitine and austalide L (see Table 2). The multiple reactions monitoring (MRM) transitions of the 16 potential metabolites were targeted and detected. Differences between the four groups was compared. The final results are provided in Fig. 8 and the ANOVA results are shown in Fig. 9. The box plot chart about the 16 differential metabolites was seen in Fig. 10. With the continuous accumulation of BMI, 5 metabolites, namely ganoderiol H, LPI (18:3), sedoheptulose 1,7-bisphosphate, 2- {[hydroxyl (3-hydroxy-4-methoxyphenyl) methylidene] amino} acetic acid and austalide L, were up-regulated in the four groups, while 5 metabolites of 1-phenyl-1,3-elcosanedione, retinol acetate, p-cresol sulfate, setariol, and arachidonyl carnitine were down-regulated in the four groups. In addition, 6 metabolites with no specific variation rules, such as 2,3-Diphosphoglyceric acid, 13,14-dihydroretinol, 5-hexyl-2-furanoctanoic acid, cholesteryl sulfate, progesterone and uric acid have been found.

**Table 2:** Characterization of the biomarkers between underweight, normal weight, overweight, and obese group in follicular fluid.

Compound	m/z	Polarity	T_R_ (min)	Metabolites	P value	Pathway
M1	347.2620	−	8.66	13,14-dihydroretinol	0.00838	Retinol metabolism
M2	327.2285	−	8.62	Retinol acetate	0.00985	Retinol metabolism
M3	265.1458	−	6.89	2,3-Diphosphoglyceric acid	0.00957	Glycolysis or gluconeogenesis
M4	167.0201	−	1.84	uric acid	0.00189	Purine metabolism
M5	385.3047	−	11.34	1-phenyl-1,3-eicosanedione	0.00671	Fatty acid metabolism
M6	502.3912	−	10.9	Arachidonyl carnitine	0.00877	Fatty acid metabolism
M7	293.1772	−	7.49	5-Hexyl-2-furanoctanoic acid	0.00673	Fatty acid metabolism
M8	315.2341	+	7.51	Progesterone	0.00869	Steroid hormone biosynthesis
M9	465.2899	−	9.66	Cholesteryl sulfate	0.00061	Steroid hormone biosynthesis
M10	187.0087	−	4.01	p-Cresol sulfate	0.00714	N/A
M11	593.4691	−	11.87	LPI (18:3)	0.00376	N/A
M12	369.1727	−	4.67	Sedoheptulose 1,7-bisphosphate	0.00093	N/A
M13	224.0637	−	3.82	2-{[hydroxyl(3-hydroxy-4-methoxyphenylmethylidene]amino}acetic acid	0.00067	N/A
M14	413.3028	−	8.82	Setariol	0.030383	N/A
M15	489.3556	−	7.87	Ganoderiol H	0.00026	N/A
M16	427.2083	−	5.27	Austalide L	0.000007	N/A

### Pathway analysis

**Figure 11:**
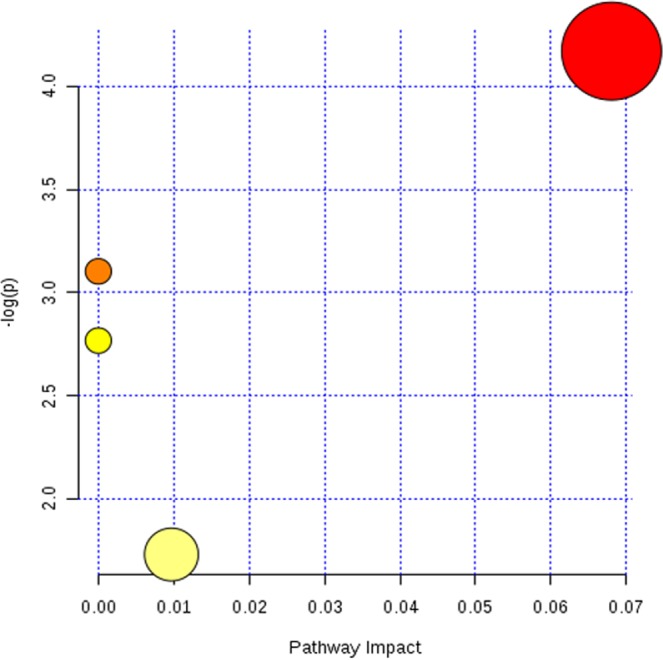
Pathway analysis diagram (red: steroid hormone biosynthesis; Pale yellow: purine metabolism, yellow: glycolysis or gluconeogenesis, orange: retinol metabolism).

MetaboAnalyst was employed to perform metabolic pathway analysis. Abnormal changes were detected in 5 metabolic pathways, i.e., steroid hormone biosynthesis, retinol metabolism, glycolysis or gluconeogenesis, and purine metabolism (see Fig. 11). In these pathways, three metabolites; retinol acetate, 1-phenyl-1,3-elcosanedione, and arachidonyl carnitine were down-regulated, while no significant changes in the metabolic pathways were observed for the up-regulated metabolites.

## Discussion

The results of this study show that being obesity in women alters the metabolic characteristics of human FF as profiled through untargeted metabolomics using the SWATH to MRM method. This method is characterized by high coverage, high sensitivity, good reproducibility, and a wide dynamic range. In this study, sixteen FF metabolites were identified and enriched in in 4 metabolic pathways including steroid hormone biosynthesis, retinol metabolism, glycolysis or gluconeogenesis, and purine metabolism. Several studies have used metabolomics to study the pregnancy outcomes of women undergoing IVF^1,32,33^. However, only a few researchers have studied the effects of obesity on FF metabolomics^34–36^. In this study, all FF of underweight, normal weight, overweight and obese women receiving IVF treatment were included into the metabolomics study. We found that the contents of retinol acetate, 1-phenyl-1,3-elcosanedione and Arachidonyl carnitine in FF decreased significantly as the BMI increased. These metabolites were involved in different metabolic pathways of retinol metabolism and fatty acid metabolism (see Table 2 and Fig. 11).

Retinol acetate (retinyl acetate, vitamin A acetate) is a natural form of vitamin A which is the acetate ester of retinol. It possesses antineoplastic and chemopreventive activities^37^. Furthermore, vitamin A and its physiological metabolites, collectively known as retinoids play essential roles in embryonic morphogenesis and reproductive physiology as a mitogenic and differentiation stimuli^38^. Retinoids induce cellular differentiation *in vitro* by modulating the expression of homeobox genes, growth factors and their receptors^39^. Previous studies have shown that retinoids can synchronize cellular events that trigger oocyte maturation, increase oocyte ability to fertilized and facilitate preimplantation embryonic development^40–43^. Cumulus cells are reported to play a dominant role in mediating the effects of retinoids during *in vitro* oocyte maturation. Retinoid-treated cumulus cells expressed lower levels of initiator and effector caspases, such as TNF-α, TNFR1, BAX^44–46^ and higher levels of BCL-2^44^. The retinoid treatment has been shown to reduce the incidence of oocyte apoptosis by diminishing apoptosis markers^44–47^. In this study, results show that the number of usable embryos obtained from overweight and obese women and the number of FET cycles in such a population was significantly smaller compared to that of women with normal BMI. Despite the fact that this study was underpowered to detect differences in CPRs, PLRs, and LBRs, a negative relationship was observed between BMI and these parameters We inferred that significantly lower levels of FF retinol acetate in overweight and obese individuals may explain this negative relationship.

Arachidonyl carnitine and 1-phenyl-1,3-elcosanedione, both of which are products of fatty acid metabolism, were also found to be significantly lower in overweight and obese women. Fatty acids, which regulate oocyte developmental competence, are stored intracellularly as triacylglycerides in lipid droplets. They are the primary and potent energy source. For instance, oxidation of the fatty acid palmitate generates 106 ATP molecules compared to glucose oxidation which yields approximately 30 ATP molecules^48,49^. Evidence indicates that obesity may compromise mitochondrial metabolism, the main energy-supplying organelles of oocytes^50–53^. Indeed, increased BMI was associated with raised levels of FF triglyceride in women undergoing IVF treatment^20,54^. Studies have demonstrated that increased FF triglyceride levels are associated with failure of oocytes to cleave and decrease the number of usable embryos^20,32^. Similar results were obtained in this study. The decrease in 1-phenyl-1,3-elcosanedione and arachidonyl carnitine in FF was related to the excess fatty acid environment and impaired mitochondrial metabolism in overweight or obese women. This adversely affected the developmental potential of the oocytes.

As the BMI increased, 7 types of metabolites were altered, but this was not the case in the corresponding metabolic pathways. Of these, 5 metabolites, ganoderiol H, LPI (18:3), sedoheptulose 1,7-bisphosphate, 2- {[hydroxyl (3-hydroxy-4-methoxyphenyl) methylidene] amino} acetic acid and austalide L, were up-regulated, while 2 metabolites, p-cresol sulfate and setariol were down-regulated.

Ganoderiol H is a metabolite of Ganoderma lucidum (reishi). El-mekkawy *et al*. found that Ganoderiol (specifically Ganoderiol F) exerted anti-HIV activity^55,56^. And, LPI (18:3) is a lysophosphatidylinositol derived from hydrolysis of phosphatidylinositol (PI). It is involved in many physiological activities in adipose tissues, including reproduction, angiogenesis, apoptosis, and inflammation^57^. Recent studies show that the LPI/GPR55 system is a novel target for obesity with both normal or impaired glucose tolerance and type 2 diabetes, and a significant increase in circulating plasma LPI levels was observed in obese individuals^58^. However, the specific role of LPI in obese infertile population is not known.

Sedoheptulose 1,7-bisphosphate belongs to the class of organic compounds known as monosaccharide phosphates. These are monosaccharides comprising a phosphated group linked to the carbohydrate unit. 2-{[hydroxy(3-hydroxy-4-methoxyphenyl) methylidene] amino} acetic acid is a predicted metabolite generated by BioTransformer¹ that is produced from the metabolism of 3-hydroxy-4-methoxybenzoic acid. It is generated by glycine N-acyltransferase (Q6IB77) enzyme via a glycination-of-aryl-acid reaction. This glycination-of-aryl-acid occurs in humans. Austalide L is a mycotoxin produced by *aspergillus ustu*. Setariol is found in cereals and cereal products. Setariol is a constituent of the leaves of *setaria italica* (foxtail millet). p-Cresol sulfate is a microbial metabolite that is found in urine and likely derives from secondary metabolism of p-cresol. p-Cresol sulfate is the major component of urinary myelin basic protein-like material (MBPLM)^59^. It appears to be elevated in the urine of individuals with progressive multiple sclerosis^60^. It has also been linked to cardiovascular disease and oxidative injury^59^. Some of the metabolites described above have been found in some diseases, while others are rarely reported. Thus, their roles in obese infertile patients remain obscure.

This study has the following limitations. All the participants were exposed to similar fertility procedures and stimulations. The FF was collected by the same physician, in the same laboratory, and processed by the same individual, to rule out possible potential effects of interpatient variability. Our results reflect the differences in obesity. Majority of studies on the effects of obesity on the FF milieu are biased by the fact that obese women usually require higher doses of gonadotropins for stimulation. In this study, there was no significant difference in gonadotropin dosages and interval of COS among the four groups, and thus the bias caused by medication effects on the FF milieu did not exist. Nevertheless, we collected pooled FF from multiple follicles as it is the standardized procedure in our reproductive center. Another limitation is the small sample size (n = 160), although it is still larger than in most previous studies on this topic^19,34,35,54^.

## Conclusions

In conclusion, this study demonstrates significant alterations in the FF microenvironment of obese women undergoing IVF treatment, which may contribute to reduced fertility. The results of this study imply that the FF microenvironment of obese women may increase oocyte apoptosis and mitochondrial dysfunction. The extent to which these changes affect oocyte quality, maturation, fertilization potential, and embryonic development is not clear. Furthermore, appropriate measures, especially lifestyle interventions, which are likely to be constructive for obese women undergoing IVF treatment on their pregnancy outcomes, can be taken in the future. Nevertheless, this still needs to be explored in further clinical trials^19,54^.
